# Uromodulin Regulates Murine Aquaporin−2 Activity via Thick Ascending Limb–Collecting Duct Cross−Talk during Water Deprivation

**DOI:** 10.3390/ijms23169410

**Published:** 2022-08-20

**Authors:** Tomoaki Takata, Shintaro Hamada, Yukari Mae, Takuji Iyama, Ryohei Ogihara, Misako Seno, Kazuomi Nakamura, Miki Takata, Takaaki Sugihara, Hajime Isomoto

**Affiliations:** 1 Division of Gastroenterology and Nephrology, Faculty of Medicine, Tottori University, Yonago 683-8504, Japan; 2 Advanced Medicine & Translational Research Center, Organization for Research Initiative and Promotion, Tottori University, Yonago 683-8504, Japan; 3 Advanced Medicine, Innovation and Clinical Research Center, Tottori University Hospital, Yonago 683-8504, Japan; 4 Division of Respiratory Medicine and Rheumatology, Faculty of Medicine, Tottori University, Yonago 683-8504, Japan

**Keywords:** aquaporin−2, collecting duct, diuretics, endocytosis, hypertension, loop of Henle, thick ascending limb, uromodulin, vasopressin, water channel

## Abstract

Uromodulin, a urinary protein synthesized and secreted from the thick ascending limb (TAL) of the loop of Henle, is associated with hypertension through the activation of sodium reabsorption in the TAL. Uromodulin is a potential target for hypertension treatment via natriuresis. However, its biological function in epithelial cells of the distal nephron segment, particularly the collecting duct, remains unknown. Herein, we examined the regulation of uromodulin production during water deprivation in vivo as well as the effect of uromodulin on the activity of the water channel aquaporin−2 (AQP2) in vitro and in vivo using transgenic mice. Water deprivation upregulated uromodulin production; immunofluorescence experiments revealed uromodulin adhesion on the apical surface of the collecting duct. Furthermore, the activation of AQP2 was attenuated in mice lacking uromodulin. Uromodulin enhanced the phosphorylation and apical trafficking of AQP2 in mouse collecting duct cells treated with the vasopressin analog dDAVP. The uromodulin-induced apical trafficking of AQP2 was attenuated via endocytosis inhibitor treatment, suggesting that uromodulin activates AQP2 through the suppression of endocytosis. This study provides novel insights into the cross−talk between TAL and the collecting duct, and indicates that the modulation of uromodulin is a promising approach for diuresis and hypertension treatment.

## 1. Introduction

The kidney plays a central role in regulating homeostasis in the body. For example, extracellular fluid volume is controlled by the urinary excretion of sodium, and body fluid osmolality is controlled by urine concentration [1]. Renal tubular epithelial cells are responsible for the excretion of solutes and water. One of the most elaborate mechanisms underlying these physiological functions is the regulation of urine concentration through a countercurrent multiplier mechanism based on the osmotic gradient within the renal interstitium [2]. High osmolality generated by the reabsorption of sodium mainly through the thick ascending limb (TAL) of the loop of Henle is the driving force behind water reabsorption at the collecting duct (CD). Therefore, sodium reabsorption in the TAL and water reabsorption in the CD occur cooperatively to maintain body fluid levels. However, the detailed cross-talk between the TAL and CD remains elusive. Regarding the pathophysiology of hypertension, excess sodium and/or water reabsorption in the kidney is responsible for fluid overload, leading to elevated blood pressure [3,4]. Thus, elucidation of the mechanisms connecting the TAL and CD is important for the development of novel therapeutic agents for hypertension.

Uromodulin, also known as the Tamm−Horsfall protein, is a urinary protein exclusively expressed in the kidney [5]. Mutation in the genes encoding uromodulin (*UMOD*) is responsible for autosomal dominant tubulointerstitial kidney disease, previously termed as familial juvenile hyperuricemic nephropathy or medullary cystic kidney disease [6,7]. Uromodulin is mostly synthesized and secreted into urine from epithelial cells of the TAL [5,8]. Uromodulin is a glycosylphosphatidylinositol (GPI) −anchored protein mainly expressed in the apical surface of the TAL. Uromodulin is released into urine through proteolytic cleavage by a serine protease hepsin [8,9]. Uromodulin released in the urine forms polymers and the specific cleavage is an important step for polymerization [10]. Uromodulin has various biological functions, including protection against urinary stone formation [11,12] and urinary tract infection [13,14,15,16], and regulation of sodium reabsorption through the activation of the Na^+^–K^+^–2Cl^−^ cotransporter (NKCC2) and sodium chloride cotransporter (NCC) [17,18,19]. Moreover, TAL−secreted uromodulin reaches downstream nephron segments and regulates the activity of Ca^2+^ channels expressed on the apical surface of the distal convoluted tubule and connecting tubule [20]. TAL−secreted uromodulin may also affect epithelial cell function in the distal nephron segment, including the CD. In the CD, a water channel, aquaporin−2 (AQP2), plays a central role in regulating water reabsorption; furthermore, AQP2 activity depends on phosphorylation and apical protein abundance. Considering its site of production and its regulatory effect on remote nephron segments, uromodulin may be implicated in the cross-talk between the TAL and CD through the mediation of AQP2 activity.

In this study, we aimed to investigate the relevance of uromodulin in the regulation of water reabsorption. We examined the regulation of uromodulin production under the conditions of water deprivation in vivo. Further, we investigated the effect of uromodulin on water channel activities in vitro and in vivo using transgenic mice.

## 2. Results

### 2.1. Water Deprivation Increases the Urinary Secretion of Uromodulin

**Figure 1:**
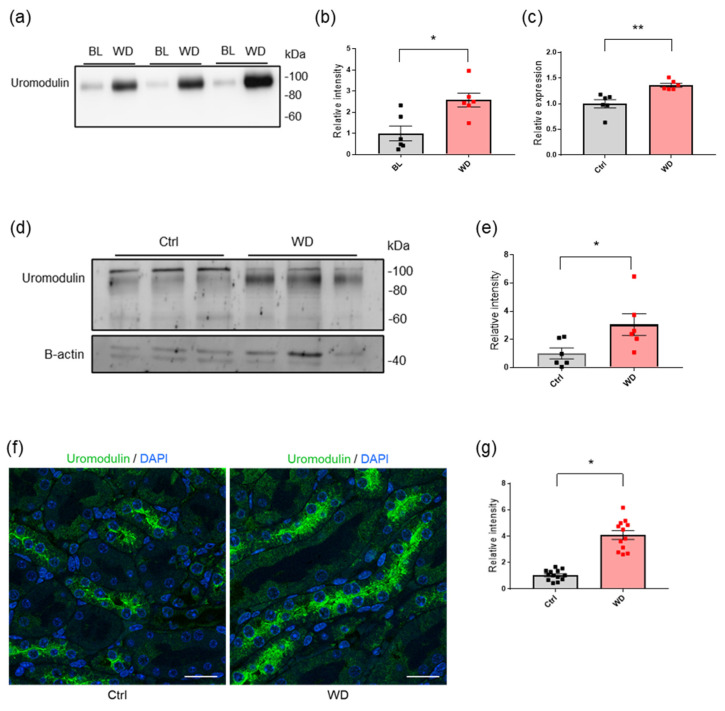
Regulation of uromodulin under water deprivation in WT mice. (**a**) Western blot analysis for urinary uromodulin at baseline (BL) and after 24 h of water deprivation (WD). Levels were normalized to urinary creatinine to account for urine concentration. (**b**) Quantification of uromodulin signals indicated that urinary uromodulin secretion significantly increased after water deprivation. *N* = 6. * *p* < 0.05 (Paired *t*-test). (**c**) Transcript levels of *Umod* analyzed via RT−PCR of whole-kidney extracts from mice with access to free water (Ctrl) or under water deprivation. Expression levels were normalized to the housekeeping gene, *Actβ*, and were expressed relative to control. *N* = 6. ** *p* < 0.01 (Unpaired *t*-test). (**d**) Western blot analysis for kidney uromodulin from control mice and mice under WD. β−actin was used as the loading control. (**e**) Quantification of signal intensity relative to the control. *N* = 6. * *p* < 0.05 (Unpaired *t*-test). (**f**) Representative immunofluorescence images for uromodulin (green) in kidney sections from control and water−deprived mice. The scale bar indicates 20 µm. (**g**) Quantification of the ratio of uromodulin signal intensities to the tubular area. The analysis was based on at least three microscopic fields of tissue from three mice per group. * *p* < 0.05 (Unpaired *t*-test).

First, we examined the regulation of uromodulin production and secretion in wild−type (WT) mice subjected to dehydration. To eliminate the influence of urine concentration, uromodulin abundance was investigated in urine and normalized to the creatinine concentration. Urinary uromodulin secretion dramatically increased when the mice were deprived of water (Figure 1a,b). Further, we investigated the transcript and protein levels of uromodulin in the kidneys of the mice under water deprivation. Umod mRNA expression (Figure 1c) and protein levels (Figure 1d,e) were significantly higher in the kidneys of water−deprived mice than in the kidneys of mice with free access to water (*p* < 0.01 and *p* < 0.05, respectively). Our observations were further confirmed via immunofluorescence staining, which showed strong uromodulin signals in the cellular and apical membrane of the TAL (Figure 1f,g).

### 2.2. Water Deprivation and Free Water Reabsorption

**Figure 2:**
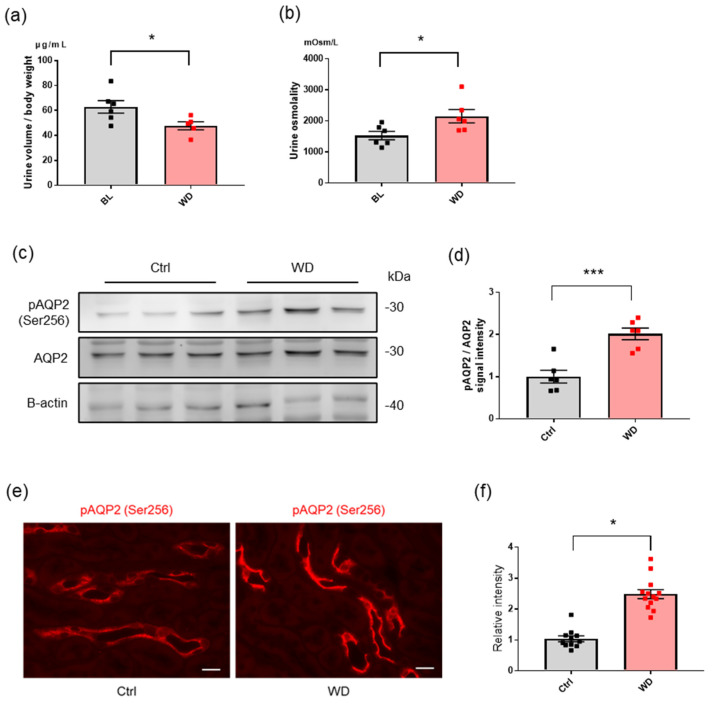
Activation of AQP2 by water deprivation. Changes in urinary volume (**a**) and urine osmolality (**b**) in mice at baseline and after 24 h of water deprivation. Each dot represents a mouse. * *p* < 0.05 (Welch’s *t*-test). (**c**) Western blot analysis for Ser256−phosphorylated and total AQP2 in kidney tissue from control mice and mice under water deprivation. β−actin is also shown as a loading control. (**d**) Quantification of the signal intensities expressed as a ratio of phosphorylated AQP2 to total AQP2. *N* = 6. *** *p* < 0.001 (Unpaired *t*-test). (**e**) Representative immunofluorescence images for Ser256−phosphorylated AQP2 (red) in kidney sections from control and water−deprived mice. The scale bar indicates 20 μm. (**f**) Quantification of the ratio of phosphorylated AQP2 signal intensities to the tubular area. The analysis was based on at least three microscopic fields of tissue from each group of mice. * *p* < 0.05 (Unpaired *t*-test).

Additionally, we confirmed that water deprivation effectively induced water reabsorption, as previously described [21]. Urinary volume significantly decreased after 24 h of water deprivation (Figure 2a) (*p* < 0.05). Moreover, urinary osmolality increased after water deprivation, indicative of upregulated free water reabsorption in the CD (Figure 2b). Water reabsorption through the CD depends on the permeability of free water via AQP2, where the translocation of AQP2 from the cytoplasmic vesicle to the apical surface and its phosphorylation of AQP2 are necessary steps [22,23,24,25]. Our physiological observations in mice were further confirmed by the increase in phosphorylated AQP2 levels in the kidneys of water-deprived mice (Figure 2c–f).

### 2.3. Uromodulin Adheres to the Apical Surface of the CD

**Figure 3:**
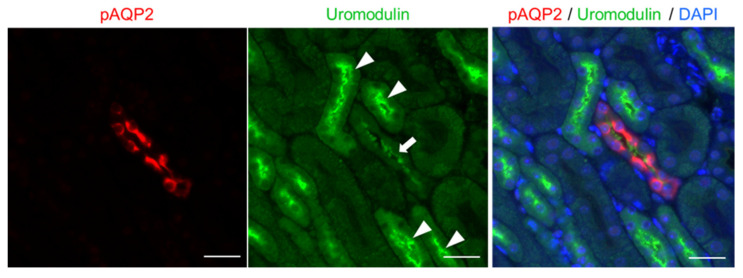
Uromodulin signals detected at the apical surface of the collecting duct. Representative immunofluorescence images for Ser256−phosphorylated AQP2 (**red**), uromodulin (**green**), and merged image with 4′,6−diamidino−2−phenylindole (DAPI) (**blue**) on Carnoy’s solution−fixed kidney sections from a wild−type mouse deprived of water for 24 h. Apical and cytosolic uromodulin signals were observed in the thick ascending limb of the loop of Henle (arrowheads). Uromodulin signals can be detected at the apical surface of the collecting duct (arrow). The scale bar indicates 20 μm.

Uromodulin forms filaments in urine and is capable of self−polymerization [26]. Owing to its structural features, uromodulin forms macromolecular structures at the epithelial surface of the distal tubule [20]. Therefore, we investigated whether uromodulin could be detected on the epithelial surface of the distal tubule. Immunostaining analysis of water−deprived mouse kidney sections fixed with 4% paraformaldehyde was performed; although the signal was faint, uromodulin could be detected at the apical surface of CD cells (Figure S1). Subsequently, we used Carnoy’s solution for immunostaining analysis. Carnoy’s solution is a precipitant fixative and preferable for the detection of large proteins; it preserves tissue architecture to a greater extent than paraformaldehyde [27]. Uromodulin was detected along the apical surface of the CD (Figure 3). Given that uromodulin synthesis and secretion were limited to the TAL and distal convoluted tubule [28], the secreted uromodulin may reach the CD and adhere to the apical surface of the epithelial cells.

### 2.4. Generation of Uromodulin Knock-Out Mice

**Figure 4:**
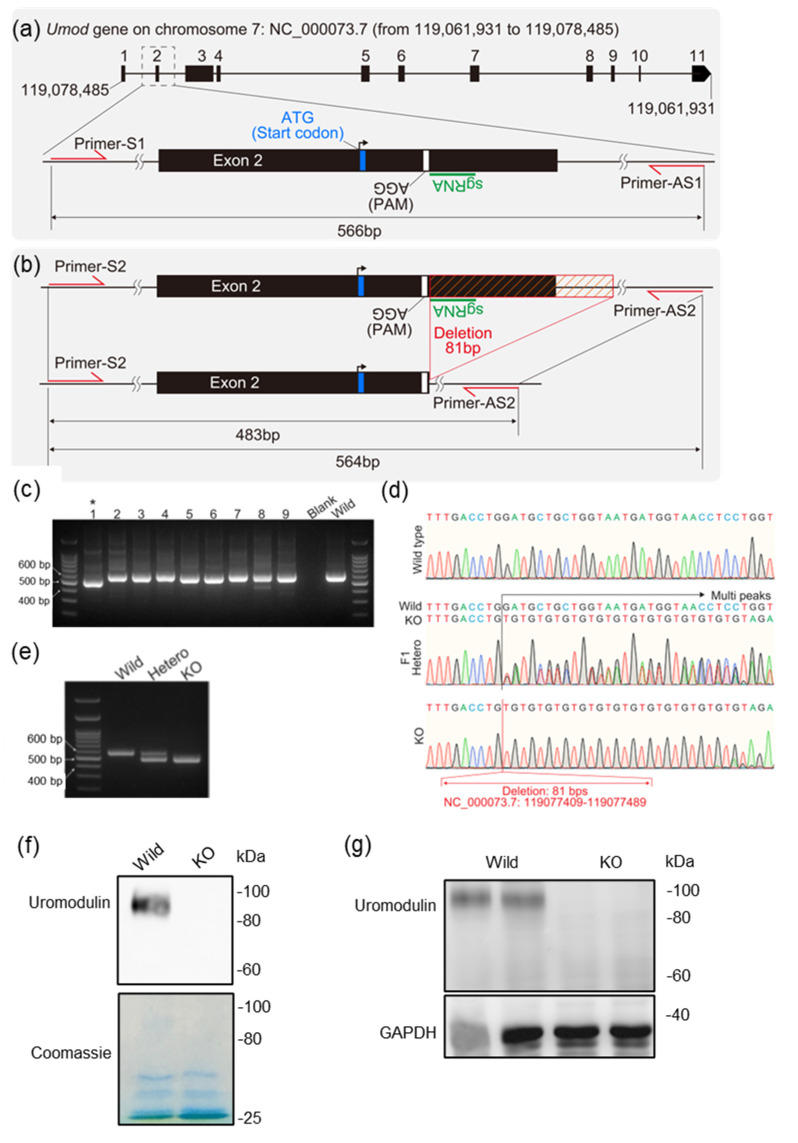
Generation of Umod−KO mice. (**a**) The *Umod* locus on chromosome 7, sgRNA-targeted region, and PCR primers are shown. (**b**) The region deleted using the CRISPR/Cas system is shown. (**c**) Electropherograms with PCR screening of F0 generation mice are shown. F0 mouse No. 1 marked with an asterisk was selected as the founder mouse. The electropherogram with the selected founder’s band is shown. (**d**) Chromatograms of sequence analysis are shown. The lengths of the horizontal axis of the chromatogram images were adjusted to the same length. Chromatograms of F1 mice show multiple peaks due to heterozygosity. Eighty−one base pairs (from 119,077,409 to 119,077,489 on NC_000073.7) were deleted using the CRISPR/Cas system. (**e**) Electropherogram of genotyping PCR is shown. Deletion of uromodulin was confirmed using western blot analysis for uromodulin in urine (**f**) and kidney samples (**g**). PAM: proto−spacer−adjacent motif.

Of the 69 mouse zygotes that were microinjected with a mixture of single guide RNA (sgRNA) and Cas9, 54 zygotes developed to the two−cell embryo stage in vitro. These were transferred to pseudopregnant mice, and 10 pups (F0 generation) were obtained. The F0 mice with shorter band sizes, compared to wild−type animals, as determined via screening PCR analysis, were selected as founder mice (Figure 4c). Sequence analysis of the F1 generation and homozygous mice revealed a deletion from 21 bp downstream of the start codon (on the second exon) to 13 bp from the 5′ end of the second intron (Figure 4b,d). The genotype of the uromodulin knock-out (Umod−KO) strain mice was validated using PCR and electrophoresis (Figure 4e). The Umod−KO mice were also confirmed to not produce uromodulin by subjecting their urine and kidney samples to western blot analyses (Figure 4f,g).

### 2.5. Uromodulin-Deficient Mice Exhibit Defective AQP2 Activation

**Figure 5:**
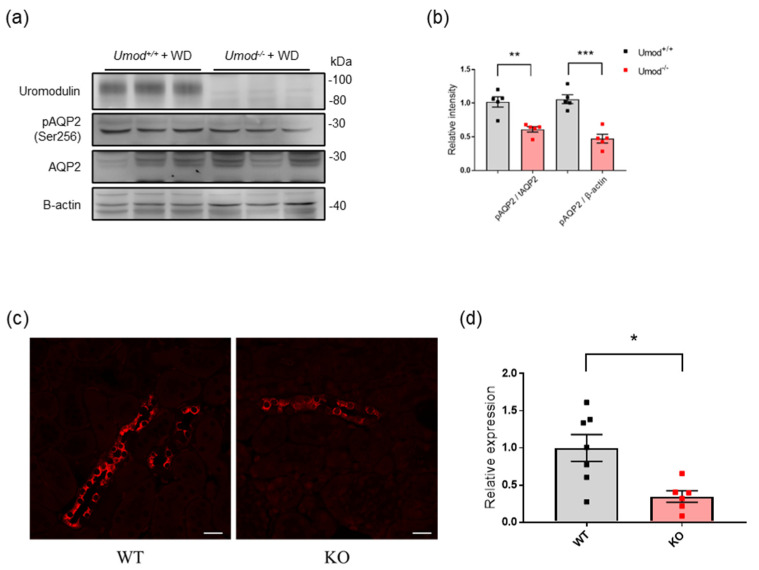
AQP2 activation is altered in uromodulin−deficient mice. (**a**) Western blot analysis for uromodulin, Ser256−phosphorylated AQP2 and total AQP2 in kidney tissue from WT and Umod−KO mice after 24 h of water deprivation. β−actin was used as a loading control. (**b**) Quantification of signal intensities expressed as the ratio of phosphorylated AQP2 to total AQP2. *n* = 6. ** *p* < 0.01, *** *p* < 0.001 (Unpaired *t*−test). (**c**) Representative immunofluorescence images for Ser256−phosphorylated AQP2 (red) in kidney sections from WT and Umod−KO mice, with strong signals in WT mouse kidney tissue. The scale bar indicates 20 μm. (**d**) Quantification of the ratio of phosphorylated AQP2 signal intensities to the tubular area. The analysis was based on at least three microscopic fields of tissue from each group of mice. * *p* < 0.05 (Unpaired *t*−test).

Furthermore, we investigated the physiological role of uromodulin in water homeostasis using uromodulin−deficient mice. We generated Umod−KO mice through the inactivation of murine *Umod*. Although the protein levels of total AQP2 did not differ between WT and Umod−KO mice after water deprivation, the phosphorylation of AQP2 was attenuated in Umod−KO mice (Figure 5a,b). Immunostaining revealed that phosphorylated AQP2 signals were stronger in WT mice than in *Umod^−/−^* mice following water deprivation (Figure 5c).

### 2.6. Effect of Uromodulin on AQP2 Phosphorylation in Mouse CD Cells

**Figure 6:**
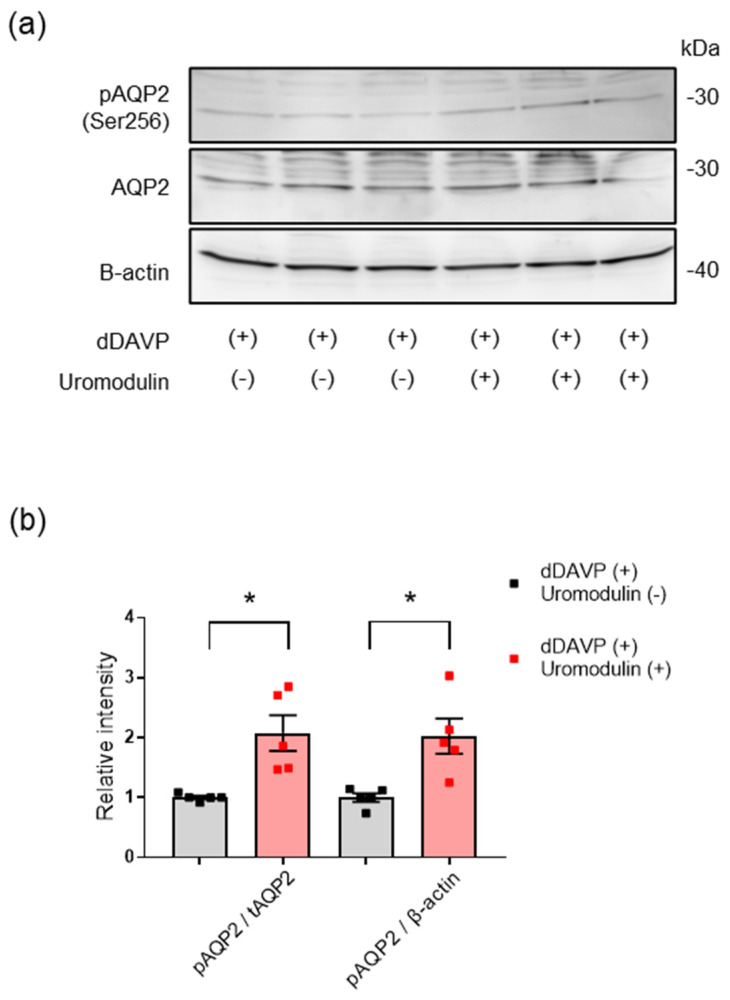
Effect of uromodulin on AQP2 activation. (**a**) Western blot analysis for Ser256-phosphorylated AQP2 and total AQP2 in lysates of mouse collecting duct cells. Cell monolayer on a filter membrane was treated with 10^−9^ M dDAVP on the basolateral side and 10^−6^ µg/mL uromodulin on the apical side for 24 h. β-actin was used as a loading control. (**b**) Quantification of the signal intensities expressed as a ratio of phosphorylated AQP2 to total AQP2. Analyses were based on at least three independent experiments. * *p* < 0.05 (Unpaired *t*-test).

Vasopressin mediates the transcription, phosphorylation, and membrane trafficking of AQP2 [29,30]. The binding of vasopressin to its receptor (V2R) in the basolateral membrane of the collecting duct cell stimulates cAMP signaling and increases the transcription and phosphorylation of AQP2 [31,32]. To investigate the effect of uromodulin on vasopressin−induced AQP2 activation, mouse CD cells were treated with the V2 receptor−specific vasopressin analog dDAVP, with or without uromodulin. Effective dDAVP treatment of CD cells was confirmed by the increase in cAMP levels and AQP2 phosphorylation; 10^−9^ M dDAVP was used in subsequent experiments (Figure S2). Apical uromodulin enhanced the phosphorylation of AQP2 in CD cells treated with dDAVP (Figure 6a,b).

### 2.7. Uromodulin May Activate AQP2 through the Suppression of Endocytosis

**Figure 7:**
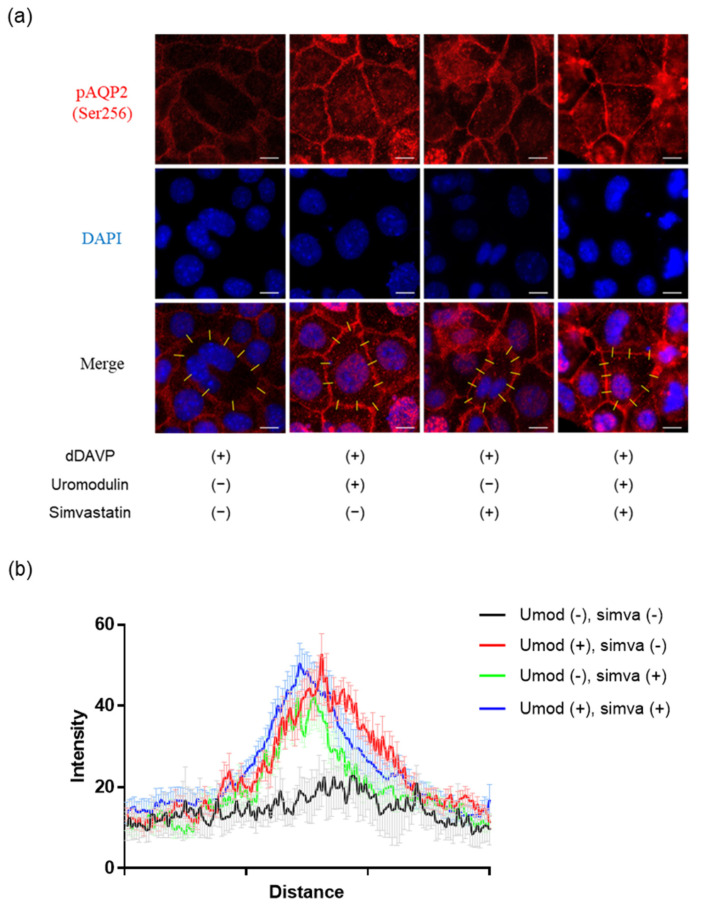
Uromodulin may activate AQP2 through the suppression of endocytosis. (**a**) Representative immunofluorescence images for Ser256−phosphorylated AQP2 in mouse collecting duct cells treated with 10^−9^ M dDAVP with or without 10^−6^ µg/mL uromodulin and 100 µM simvastatin for 24 h. Uromodulin-treated cells showing strong signals for phosphorylated AQP2 (red). Strong linear signals for phosphorylated AQP2 at the membrane can be observed in cells treated with simvastatin, regardless of uromodulin treatment. (**b**) Intensity profiles for Ser256-phosphorylated AQP2 along axis across the cell membrane. Quantifications are based on ten axes (yellow lines) in each condition and showed as mean and SEM. Bars indicate 10 μm. DAPI, 4’,6−diamidino−2−phenylindole; umod, uromodulin; simva, simvastatin.

AQP2 is a protein that is subjected to constant recycling, and its apical trafficking is regulated through vasopressin−modulated endocytosis and exocytosis [25,29,33]. As uromodulin has an inhibitory effect on the endocytosis of membrane transporters [20], we investigated whether uromodulin affects the membrane expression of AQP2 in CD cells. Uromodulin enhanced the expression of phosphorylated AQP2 in vasopressin−stimulated CD cells (Figure 7a). Furthermore, we examined the effect of uromodulin on apical AQP2 trafficking under treatment with the endocytosis inhibitor simvastatin over a duration and at concentrations that have been previously reported [34]. Uromodulin treatment in combination with simvastatin did not increase the apical expression of phosphorylated AQP2 as much as without simvastatin (Figure 7b). These results indicated that uromodulin upregulates the apical expression of phosphorylated AQP2 through the suppression of endocytosis.

## 3. Discussion

In this study, we demonstrated that urinary and renal uromodulin levels are upregulated under dehydration in vivo. In addition, we showed that uromodulin adhered to the apical surface of the CD. Further, uromodulin promoted AQP2 activity in vivo as well as in CD cells. These findings suggest that uromodulin is involved in water homeostasis through the mediation of cross−talk between the TAL and CD.

Uromodulin regulates sodium reabsorption by activating relevant transporters, including NKCC2 and NCC [28,35]. In particular, sodium reabsorption through NKCC2 plays an important role in maintaining osmolality within the interstitium, driving the passive transport of free water via AQP2 [1]. Therefore, increased urinary and renal uromodulin levels may be a reasonable response to water deprivation. A previous report demonstrated that mice under short−term treatment with vasopressin, a hormone synthesized in response to dehydration, exhibited increased urinary uromodulin secretion together with reduced intracellular uromodulin levels [36]. This is, in part, consistent with our results. However, we observed increased transcript and protein levels of uromodulin. A possible reason for this discrepancy is the difference in the experimental setup. We used a mouse model of dehydration, whereas the previous study employed a model of water overload with vasopressin. Thus, serum osmolality may regulate uromodulin production. In fact, NaCl overload, which leads to high serum osmolality, increased urinary uromodulin without any deficit in renal uromodulin levels [37]. Furthermore, transgenic mice lacking the ability to secrete uromodulin exhibited a massive increase in intracellular uromodulin levels without any increase in urinary uromodulin levels following NaCl overload [37]. These findings indicate that the synthesis and secretion of uromodulin may be differently regulated.

In addition to its functions in the TAL, uromodulin is associated with cast formation in multiple myeloma, protection against urinary stones, and protection against urinary tract infections [38,39]. Urinary uromodulin forms a gel−like structure with important physiological functions, including the adhesion to uropathogenic *Escherichia coli* [14]. In this study, we employed Carnoy’s solution instead of paraformaldehyde for tissue fixation. This change enabled us, for the first time, to observe urinary uromodulin adhesion to the apical surface of the distal nephron segment. In addition, uromodulin−deficient mice in our study exhibited lower AQP2 activity. These findings indicate that intraluminal uromodulin secreted from the TAL reaches to the downstream segment and influences the epithelial cell membrane function of the CD. In order to confirm our hypothesis, in vitro studies using CD cells were conducted. Uromodulin was added to the apical side of the monolayer of cell sheet, then AQP2 activity and localization was investigated. The activity of AQP2 depends on its phosphorylation and trafficking to the apical membrane surface [40]. Uromodulin upregulated AQP2 phosphorylation and the apical expression of phosphorylated AQP2, possibly through the inhibition of endocytosis, as previously suggested [20]. AQP2 on the apical surface of CD cells determines water permeability and thus the final concentration of urine [41]. Therefore, uromodulin is involved in the regulation of urine concentration. Although we demonstrated that uromodulin has an effect on AQP2 activity, its effect of AQP2 upregulation relative to vasopressin was not demonstrated in our study. We observed that uromodulin−deficient mice expressed phosphorylated AQP2 to some extent. This suggests that uromodulin is not the dominant molecule regulating AQP2 activity. However, in vivo experiments revealed that the absence of uromodulin has certain influence on the expression of phosphorylated AQP2 under water dehydration. In addition, in vitro experiments showed that uromodulin treatment has influenced the phosphorylation and localization of AQP2 in vasopressin–treated CD cells. These results indicate that uromodulin has an additive effect on AQP2 activation.

In this study, we demonstrated that uromodulin is involved in the regulation of AQP2. Our findings suggest that uromodulin may upregulate AQP2 through inhibition of endocytosis, however detailed mechanisms of the uromodulin–AQP2 interaction is not elucidated. Similar to membrane transporters such as ATP−binding cassette transporters and solute carrier transporters, the activity of water channel AQPs is regulated through various mechanisms including trafficking, gating, and protein–protein interactions [42,43,44]. Among them, trafficking to the membrane and endocytosis are the most common regulatory mechanisms, particularly for AQP2 [44]. As described above, vasopressin–mediated cAMP induction triggers AQP2 trafficking to the apical surface [31,32]. A number of proteins have been identified that affect this process. These include c heat shock protein 70, annexin II, LYST–interacting protein, and ezrin [45,46,47]; some of them interact with AQP2 via post–translational modification. Although further study is necessary to uncover the mechanism, uromodulin may interact with AQP2 in a post–translational manner.

We demonstrated that uromodulin production is upregulated by the loss of body fluids and that uromodulin, in turn, modulates urine concentration through the activation of AQP2. The regulation of uromodulin in response to the change in the balance of body fluids may be associated with resistance to diuretics or anti−hypertensive agents. Therefore, it is speculated that the downregulation of uromodulin production or the inhibition of its adhesion to the apical surface of the CD may suppress AQP2 activity, leading to diuresis or lowering of blood pressure. Although further investigations are required with the use of diuretics or under a hypertensive condition, our study provides new insights into the TAL–CD cross−talk via uromodulin.

## 4. Materials and Methods

### 4.1. Generation of Knock−Out Mice

Umod–KO mice were generated using CRISPR/Cas technology. The sequence of the sgRNA targeting the mouse uromodulin gene (Umod: NC_000073.7), designed by Macrogen (Seoul, Korea), was 5′−CATCATTACCAGCAGCATCC−3′ (Figure 1a,b). Cas9 nuclease was purchased from Integrated DNA Technologies (Alt-R S.p. Cas9 Nuclease V3, Integrated DNA Technologies, Inc., Coralville, IA, USA). Cas9 and sgRNA were mixed and diluted with water (W1503, water for embryo transfer, sterile−filtered, BioXtra, suitable for mouse embryo, Sigma−Aldrich Co., St. Louis, MO, USA). The final concentrations of Cas9 and sgRNA were 20 ng/µL and 10 ng/µL, respectively. The mixtures were microinjected into the pronuclei of fertilized zygotes collected from C57BL/6J mice. These zygotes were developed into two-cell embryos in vitro and transferred into the oviducts of pseudopregnant ICR mice. Genomic PCR was used to screen for founder (F0) mice carrying the desired mutation. F1 mice were obtained by mating male F0 mice and female C57BL/6J mice. The genome of F1 mice was sequenced using genomic PCR by Eurofins Genomics K.K. (Tokyo, Japan). Homozygous (Umod–KO) mice were obtained by mating males and females with the same mutated allele, and the mutated allele sequences were determined by comparing the sequence data with the reference genome sequence (NC_000073.7) in the NCBI database. Primers S1 (sense) and AS1 (anti-sense) were used for genomic PCR of F0, whereas S2 and AS2 were used for the others (Figure 1a,b). The sequences of primers S1, AS1, S2, and AS2 were 5′−AAGCTGTGCCTCCTGGTCTA−3′, 5′−TTGTCCCACCAGAAACATCA−3′, 5′−TGTGCCTCCTGGTCTACATCCATG−3′, and 5′−CCTTGTCCCACCAGAAACATCACC−3′, respectively. The sense primers were also used for sequence analysis. C57BL/6J and ICR mice were purchased from Japan SLC (Hamamatsu, Japan).

### 4.2. Experimental Protocol

Eight−week−old male mice were used for water deprivation experiments. The mice were housed under 12 h light–dark cycles with ad libitum access to water and standard pellet chow (CE−2; CLEA Japan, Tokyo, Japan). Mice were individually housed in metabolic cages for 24 h for the collection of urine to determine baseline conditions. The second metabolic studies for 24 h urine collection were performed one week after the first metabolic studies in two randomly assigned groups: one with free access to water (*n* = 6) or one without free access to water (*n* = 6). Urine was briefly centrifuged and stored at −80 °C until analysis. Mice were anesthetized with medetomidine, midazolam, and butorphanol. Blood was obtained from the heart and stored at −80 °C after centrifugation for 15 min at 2000× *g*. At the end of the second metabolic studies, mice were sacrificed by cervical dislocation, and the harvested kidneys were immediately frozen in liquid nitrogen and stored at −80 °C until analysis.

### 4.3. Cell Culture and Treatment

Mouse cortical CD cells (M−1, KAC Inc., Kyoto, Japan) were purchased for in vitro studies. The cell line was maintained in Dulbecco’s modified Eagle medium/Ham’s F−12 (048−29785; FUJIFILM Wako Pure Chemical Co., Osaka, Japan) supplemented with 5 μM dexamethasone (047−18863; FUJIFILM Wako Pure Chemical Co., Osaka, Japan) and 5% fetal bovine serum (FB−1356; FUJIFILM Wako Pure Chemical Co., Osaka, Japan) in a humidified incubator with 5% CO_2_ at 37 °C. When near confluence, cells were passaged to a 0.33 cm^2^ PTFE filter membrane (Corning, Shizuoka, Japan) using 0.05% trypsin/EDTA. A confluent monolayer of the cells was maintained in serum-free and hormone−deprived medium for 24 h and then used for the experiment [48]. Cells were treated with the V2 receptor−specific vasopressin analog dDAVP on the basolateral side (17748; Cayman Chemical, Ann Arbor, MI, USA), with or without mouse recombinant uromodulin (LS−G15802; LSBio, Seattle, WA, USA) to the apical side for 24 h. For the analysis of dDAVP−induced cAMP production, cells were cultured in a 96−well plate and treated with dDAVP for 24 h. cAMP concentrations were measured using cAMP−Glo assays (V1501; Promega, Tokyo, Japan) according to the manufacturer’s protocol. For the analysis of endocytosis inhibition, cells were passed into a fibronectin−coated slide−chamber. Confluent cells were treated with dDAVP and uromodulin for 24 h, followed by 1 h treatment with 100 µM simvastatin (AG−CN2−0052; Funakoshi, Tokyo, Japan). Cells were then fixed and subjected to immunofluorescence analysis.

### 4.4. Reverse Transcriptase−Polymerase Chain Reaction (RT−PCR) Analysis

Gene expression was analyzed as previously described [49]. Total RNA was extracted from homogenized kidneys using the RNeasy Mini Kit (Qiagen, Hilden, Germany). Reverse transcription was performed using 2 µg DNA with the High−Capacity cDNA Reverse Transcription Kit (Thermo Fisher Scientific Inc., Tokyo, Japan). Transcript levels were measured using quantitative RT−PCR, with 0.5 µM of primers and 4 μL of LightCycler FastStart DNA Master PLUS SYBR−Green (Roche Diagnostics, Tokyo, Japan) at a final volume of 20 µL and the LightCycler 1.5 complete system (Roche Diagnostics). Primers used for *Umod* in the RT−PCR analysis were as follows: F, 5′−TTGCGAAGAATGCAGGGTAG−3′ and R, 5′−TGGCACTTTCTGAGGGACAT−3′. Primers used for *Aqp2* were as follows: F, 5′−TCACTGGGTCTTCTGGATCG−3′ and R, 5′−CGTTCCTCCCAGTCAGTGT−3′. Beta-actin (*Actb*) was used as the internal control for the normalization of expression data, with the following primers: F, 5′−AAATCTGGCACCACACCTTC−3′ and R: 5′−GGGGTGTTGAAGGTCTCAAA−3′. PCR conditions were 95 °C for 10 min, followed by 45 cycles of 10 s at 95 °C, 10 s at 60 °C, and 10 s at 70 °C. The cycle passing threshold (Ct) was recorded using LightCycler software (Roche Diagnostics).

### 4.5. Western Blot Analysis

Frozen kidneys were ground in a solution containing 0.32 M sucrose, 50 mM Tris−Hepes, 1 µM EDTA, as well as protease and phosphatase inhibitor (Roche Diagnostics). Protein concentrations were then measured using Piece 660 nm Protein Assay Reagent (Thermo Fisher Scientific). Cells were lysed in the same solution. Samples were mixed with Laemmli buffer containing 2−mercaptoethanol. Thirty micrograms of protein were loaded for sodium dodecyl sulfate-polyacrylamide gel electrophoresis and transferred to nitrocellulose membranes. After blocking with 5% skim milk or 3% bovine serum albumin in tris−buffered saline, the membranes were incubated with sheep anti−uromodulin (1:2000; Meridian Life Science, Cincinnati, OH, USA), rabbit anti−AQP2 (1:1000; Bioss, Woburn, MA, USA), rabbit anti−phospho−Ser256 AQP2 (1:1000; Aviva Systems Biology, San Diego, CA, USA), rabbit anti−GAPDH (1:2000; CST, Tokyo, Japan), or rabbit anti−β−actin (1:2000; CST, Tokyo, Japan) antibodies overnight at 4 °C. Membranes were washed with Tris-buffered saline and incubated with their corresponding secondary antibodies conjugated with horseradish peroxidase (1:2000). Coomassie blue staining was performed using InstantBlue Coomassie Protein Stain (Abcam, Tokyo, Japan). Signals were detected using Clarity Western ECL substrate (Bio−Rad, Tokyo, Japan) and an image analyzer (LAS−3000 mini; Fujifilm, Tokyo, Japan). Densitometry analysis was performed to compare signal intensities using ImageJ software 1.52a (National Institute of Health, Bethesda, MD, USA), as previously described [50].

### 4.6. Immunostaining Analysis

Harvested mouse kidneys were fixed with 4% paraformaldehyde and embedded in paraffin (FUJIFILM Wako Pure Chemical Co., Osaka, Japan). For the intraluminal uromodulin localization experiment, kidneys were perfused and fixed with 4% paraformaldehyde or Carnoy’s solution and then embedded in paraffin. Subsequently, 4 µm−thick kidney sections were obtained, deparaffinized, heated in 10 mM citrate buffer at 95 °C for 10 min for antigen retrieval, and blocked in a solution containing 3% bovine serum albumin, 0.05% Tween-20, 50 mM glycine, and 50 mM NH4Cl in phosphate−buffered saline. The kidney sections were incubated with sheep anti−uromodulin (1:300; Meridian Life Science, Cincinnati, OH, USA) or rabbit anti−phospho−Ser256 AQP2 (1:300; Bioss, Woburn, MA, USA) overnight at 4 °C. The sections were washed with phosphate-buffered saline, incubated with the corresponding fluorescein−conjugated secondary antibodies (1:300) for 1 h at 24 °C, and mounted in ProLong Gold antifade mounting medium (Thermo Fisher Scientific). Fluorescence images were obtained using a confocal microscope (LSM 780; Carl Zeiss, Osaka, Japan). Fluorescent signal intensities were quantified using the ImageJ software as previously described [51].

### 4.7. Statistical Analysis

All data are expressed as the mean ± SEM. A two−tailed Welch’s test was used for statistical analysis. Any *p*−values < 0.05 were considered indicative of statistical significance. GraphPad Prism (7.0. for Windows, GraphPad Software, San Diego, CA, USA) was used for the statistical analysis.

## Data Availability

Not applicable.

## Supplementary Materials

The following supporting information can be downloaded at: https://www.mdpi.com/article/10.3390/ijms23169410/s1.
