# Supplementary material for: Uromodulin Regulates Murine Aquaporin−2 Activity via Thick Ascending Limb–Collecting Duct Cross−Talk during Water Deprivation
Source: Int J Mol Sci. 2022 Aug 20;23(16):9410. doi: 10.3390/ijms23169410 (PMC9408883; doi:10.3390/ijms23169410)
Supplement: Supplementary file 1 [file ijms-23-09410-s001.zip › ijms-1824549-supplementary.pdf]

Uromodulin regulates murine aquaporin−2 activity via thick ascending limb−collecting duct cross−talk during water deprivation

Tomoaki Takata[1,*], Shintaro Hamada[1], Yukari Mae[1], Takuji Iyama[1], Ryohei Ogihara[1], Misako Seno[2], Kazuomi Nakamura[3], Miki Takata[4], Takaaki Sugihara[1], Hajime Isomoto[1]

[1] Division of Gastroenterology and Nephrology, Faculty of Medicine, Tottori University

[2] Advanced Medicine & Translational Research Center, Organization for Research Initiative and Promotion, Tottori University

[3] Advanced Medicine, Innovation and Clinical Research Center, Tottori University Hospital

[4] Division of Respiratory Medicine and Rheumatology, Faculty of Medicine, Tottori University.

Corresponding author: Tomoaki Takata

Division of Gastroenterology and Nephrology, Faculty of Medicine, Tottori University

Telephone number: +81-859-38-6527

Email address: t-takata@tottori-u.ac.jp

Supplementary materials

- **Figure S1**
- **Figure S2**

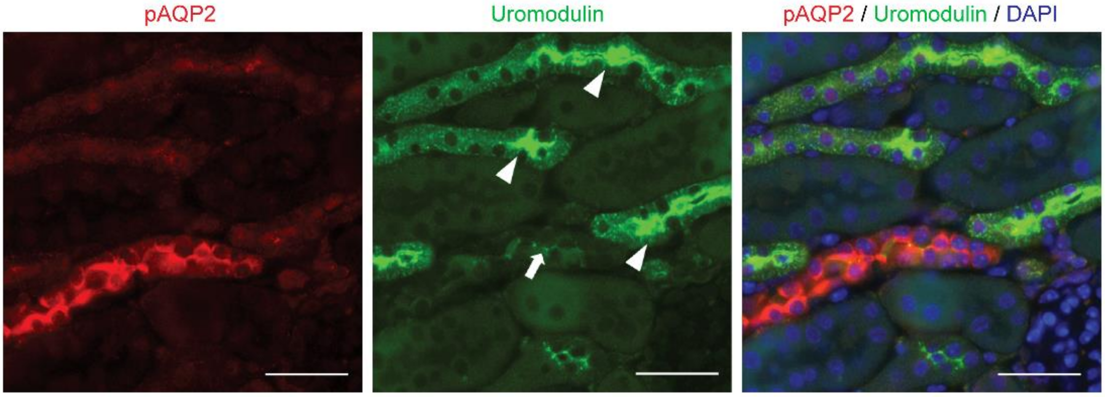

Figure S1. Uromodulin signals at the apical surface of collecting duct cells. Representative immunofluorescence images for Ser256−phosphorylated AQP2 (red), uromodulin (green), and merged image with 4′,6−diamidino−2−phenylindole (DAPI) (blue) in paraformaldehyde−fixed kidney sections from a water−deprived wild-type mouse. Apical and cytosolic uromodulin signals were observed in the thick ascending limb of the loop of Henle (arrowheads), whereas quite faint signals were noted at the apical surface of the collecting duct (arrow). Scale bar indicates 20 μm.

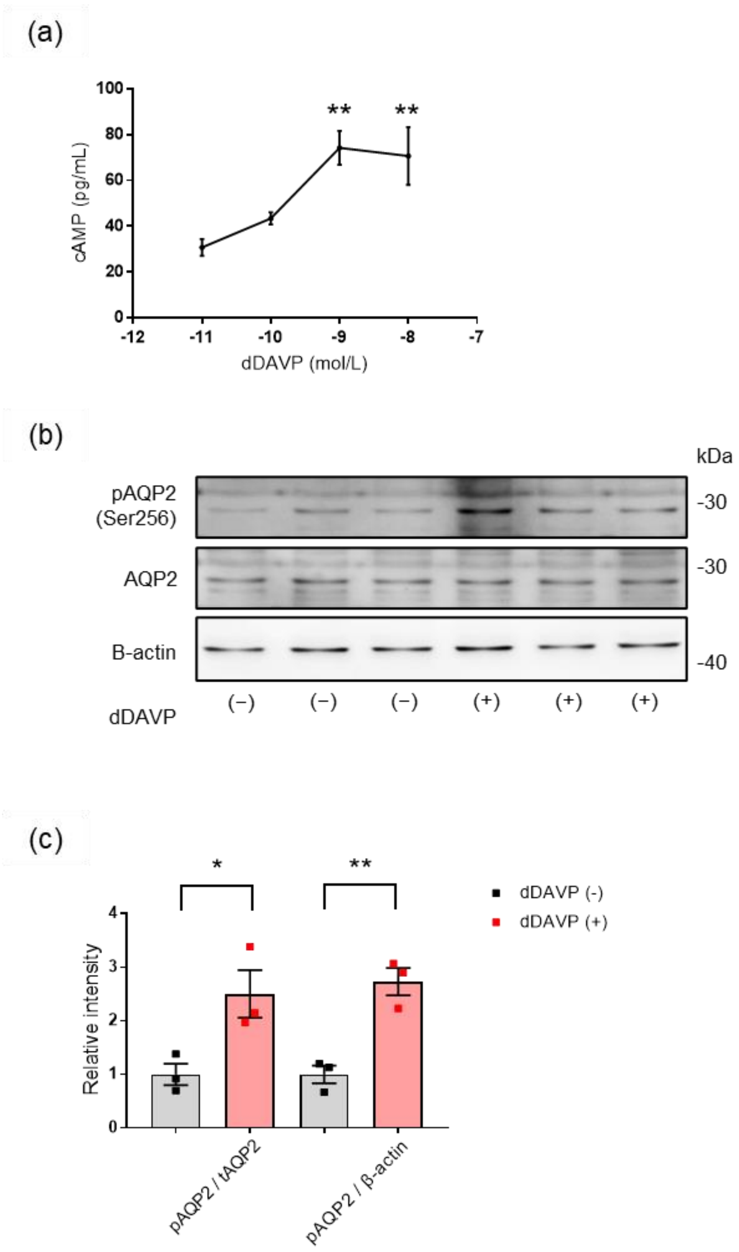

Figure S2. Activation of AQP2 by vasopressin. (a) Cellular cAMP production in mouse collecting duct cells treated with V2 receptor–specific vasopressin analog dDAVP at indicated concentrations for 24 h. Analyses were based on at least five independent experiments. **p < 0.01 (one–way analysis of variance with *post–hoc* Dunnett's test). (b) Western blot analysis for Ser256–phosphorylated AQP2 and total AQP2 in the lysate of mouse collecting duct cells. Cell monolayer on a filter membrane was treated with 10^{-9} M dDAVP at the basolateral side for 24 h. β–actin was used as a loading control. (c) Quantification of signal intensities expressed as a ratio of phosphorylated AQP2 to total AQP2. Analyses were based on at least three repetitive experiments. *p < 0.05, **p < 0.01 (Unpaired *t*–test).
